# Multicentre Prospective Study Analysing Relevant Factors Related to Marginal Bone Loss: A Two-Year Evolution

**DOI:** 10.3390/dj11080185

**Published:** 2023-07-31

**Authors:** Iñigo Fernández-Figares-Conde, Lizett Castellanos-Cosano, Juan-Alberto Fernandez-Ruiz, Ismael Soriano-Santamaria, Juan-Antonio Hueto-Madrid, Javier Gómez-Lagunas, Roberto Romano-Laureato, Daniel Torres-Lagares

**Affiliations:** 1 Department of Stomatology, School of Dentistry, University of Seville, C/Avicena s/n, 41009 Seville, Spain; fernandez_conde_94@hotmail.com; 2 Independent Researcher, Pasaje Balafi 1, 07800 Ibiza, Spain; administracion@clinicafernandez.es; 3 Independent Researcher, Calle Canarias, 7, 28045 Madrid, Spain; anabelen@formacionimplantolologica.es; 4 Independent Researcher, Pg. de la Vall d’Hebron, 119, 08035 Barcelona, Spain; hueto@iqd.es (J.-A.H.-M.); dentalcoh@gmail.com (J.G.-L.); 5 Independent Researcher, Piazza Castelnuovo, 26, 90141 Palermo, Italy; info@studioora.it

**Keywords:** marginal bone loss, dental implant, multicentre study

## Abstract

Introduction: The aim of this prospective descriptive study was to analyse the possible variables associated with marginal bone loss in rehabilitated implants (Proclinic S.A.U, Zaragoza, Spain) two years after their prosthetic loading. Materials and Methods: Three clinical centres collaborated for a period of two years after the prosthetic rehabilitation of the implants (Proclinic S.A.U, Zaragoza, Spain), in which marginal bone loss and the possible associated variables were evaluated. The collection form comprised different variables throughout different stages of the implant procedure, from implant insertion to the subsequent prosthetic rehabilitation, over a two-year period. Data of the patients and implant characteristics were studied. Statistical analysis was performed with SPSS for qualitative (univariate logistic regressions, Chi2 test, and Haberman’s corrected standardised residuals) and quantitative variables (Kolmogorov–Smirnov test). Results: The total study sample consisted of 218 implants (Proclinic S.A.U, Zaragoza, Spain). The sample presented a frequency of 99 men (45.4%) and 119 women (54.6%). The mean age of the patients among the reported cases was 58.56 ± 10.12 years. A statistically significant association was found between marginal bone loss 2 years after prosthetic rehabilitation placement and several variables, including age (under 55 years, 0.25 mm ± 0.56; 55–64 years, 0.74 mm ± 0.57; over 65 years, 0.63 mm ± 0.55; *p* < 0.0001), gender (female, 0.74 mm ± 0.61; male, 0.34 mm ± 0.51; *p* < 0.0001), bone quality (D1, 0.75 mm ± 0.62; D2, 0.43 mm ± 0.57; D3, 0.65 mm ± 0.60; *p* < 0.01), implant diameter (up to 4 mm, 0.49 mm ± 0.58; more than 4 mm, 1.21 mm ± 0.30; *p* < 0.0001), prosthetic connection type (direct to implant, 0.11 mm ± 0.58; transepithelial straight, 0.67 mm ± 0.57; transepithelial angled, 0.33 mm ± 0.25; *p* < 0001), implant model (internal conical, 0.17 mm ± 0.24; external conical, 0.48 mm ± 0.61; external cylindrical, 1.12 mm ± 0.32; *p* < 0.0001), prosthetic restoration type (full denture, 0.59 mm ± 0.59; partial denture, 0.50 mm ± 0.85; unitary crown, 0.08 mm ± 0.19; *p* < 0.05), and insertion torque (>35 N/cm, 0.53 mm ± 0.58; <35 N/cm, 1.04 mm ± 0.63; *p* < 0.01). Conclusions: At 2 years, marginal bone loss following prosthetic rehabilitation was shown to be influenced by multiple factors. Correct implantological planning is of vital importance for successful rehabilitation.

## 1. Introduction

Radiographic observation of marginal bone loss, defined as bone lost around the crestal area of the implant [1], is a criterium used for the diagnosis of peri-implant disease. Other criteria include bleeding, probing depth > 5 mm, and exposure of three or more implant coils [2,3].

Marginal bone loss around dental implants continues to be studied in detail by multiple authors, and it has been observed that it can be influenced by a multitude of factors, such as systemic factors of the patient, local factors, factors related to the implant design, the type of prosthesis, and the materials used [4,5].

The analysis of marginal bone loss has been proposed to be studied one year after the placement of the prosthetic rehabilitation and masticatory load, since during this initial period, dimensional changes ranging between 1.5 and 2 mm are established [6]. Some authors have even concluded that marginal bone loss can become progressive at around 0.2 mm per year after the first year [7].

Although radiology has seen important advances in recent years, periapical radiology is still preferred for the studying MBL, even though it would be limited to the mesial and distal aspects of the implant [8]. Articles focused on MBL that use digital orthopantomography for their study are still being published [9]. This is justified by the appearance of alterations in bone identification when CBCT is performed, which can underestimate the presence of bone in proximity to a metallic element such as the implant.

Beyond this point, studies that focus on the MBL are still fully valid to assess the different factors that influence the maintenance of peri-implant bone [10,11].

The aim of this study was to conduct a prospective multicentre study analysing marginal bone loss around placed dental implants, along with the factors associated with increased marginal bone loss 24 months after prosthetic loading.

Specifically, the following factors will be studied: gender, age, location of the implant, type of implant, diameter and length of the implant, type of bone, type of prosthetic connection, type of prosthetic rehabilitation, and insertion torque.

## 2. Materials and Methods

A prospective multicentre study was conducted to analyse the variables associated with marginal bone loss 2 years after the prosthetic rehabilitation of implants (Proclinic S.A.U, Zaragoza, Spain). We had the collaboration of three clinical centres that allowed us to access the database of the implants placed and their subsequent reviews over the course of two years. The patients included in the multicentre study were healthy patients who were not taking any medication at the time of the implant placement.

The exclusion criteria applied were as follows: patients with serious systemic diseases, such as recent heart attack, coagulation disorders, cancer, uncontrolled diabetes, psychiatric contraindications, and active infection; as well as pregnant or lactating women. Patients with pharmacological treatments capable of affecting bone healing (bisphosphonates) and gingival health, such as some anticonvulsants (phenytoin), immunosuppressants (cyclosporin A), and calcium channel blockers (nifedipine, verapamil, and diltiazem), were also excluded.

**Figure 1:**
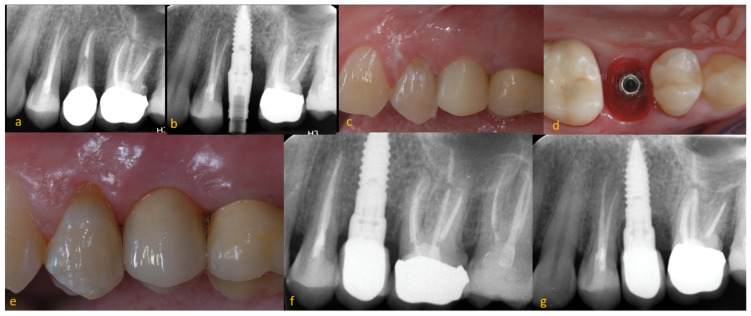
Treatment protocol applied. (**a**) Initial pre-extraction X-ray, (**b**) implant insertion, (**c**) immediate provisional crown, (**d**) peri-implant tissues, (**e**) final crown (4 months after implant placement), (**f**) X-ray of the final crown (4 months after placement implant), (**g**) X-ray of the final crown (24 months after implant placement).

In each of the collaborating centres, the implants were placed by a surgeon with more than 10 years of experience in implantology practice. The same surgical protocol for implant placement was used: infiltrative anaesthesia with articaine 1%, elevation of a mucoperiosteal flap, surgical drilling following the protocol of the commercial company according to the type of bone, implant placement and ISQ measurement (Ostell IDX, Osstell AB, Göteborg, Sweden), placement of the closure cap, and suture of the flap with supramyd 5/0 (Laboratorios Aragó, Barcelona, Spain). Analgesic (metamizole magnesium 575 mg) and antibiotic (amoxilicin 1 gr) medications were administered to all patients every eight hours for 3 days. Immediate prosthesis placement was performed in all cases within the first 24 h after implant placement, and the follow-up period began at that time, regardless of when the definitive prosthesis was made (usually four months after the operation) (Figure 1).

The variables evaluated included implant position in the maxillary or mandibular bone; type of implant placed; implant diameter; implant length; type of bone (D1, D2, D3, D4) according to Misch’s classification; type of attachment used (direct to implant, straight transepithelial, angled transepithelial, multi-unit); type of restoration (complete, partial, unitary); if the torque used was greater than 35 N/cm; if crestal expansion procedures were performed; if there was implant failure at 2 months, 6 months, and 24 months; and the cause of the failure was analysed.

Implant position was classified into four categories: maxillary anterior, including the upper central incisor (ICS), upper lateral incisor (ILS), and upper cuspid (CS); maxillary posterior, including the upper molars (MS) and upper premolars (PMS); mandibular anterior, including lower central incisor (ICI), lower lateral incisor (ILI), and lower cuspid (CI); and mandibular posterior, including lower molars (MI) and lower premolars (PMI).

**Figure 2:**
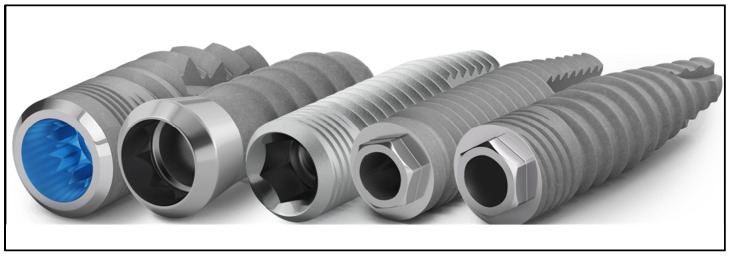
From left to right: implant M12, M8, L35, L6, and N6.

The implant design was classified into four categories: internal conical, including the M12 type implant; external conical, including the N6 type implant; internal cylindrical, including L35- and M8-type implants; and external cylindrical, including L6-type implant (Figure 2).

Marginal bone loss was measured radiographically mesially, and distally vertically from the implant crestal point of reference to the first bone-to-implant contact axially parallel to the implant at 2 months after prosthetic loading.

Marginal bone loss was evaluated at three centres by the means of periapical radiography using the parallelisation technique, which allowed for an appropriate measurement of mesial and distal marginal bone loss using Carestream Dental software (CS Imaging 8, Carestream Dental LLC, Atlanta, GA, USA). Only one person conducted the measurements at each centre, and this person received prior qualification training in relation to the program used and the measurement methodology.

The MBL measurement was performed using the implants present in the radiograph to calibrate it, thus obtaining a real measurement of the mesial and distal bone loss of the implants. This methodology has already been applied in many studies, such as ones by Sargolzaieet al. [8] and Stacchi et al. [10].

### 2.1. Ethical Considerations

This study was conducted following the ethical principles for medical research on humans as defined by the Declaration of Helsinki and following the standards of good clinical practice. Approval was obtained from the Ethical Committee of Virgen del Rocío and Virgen Macarena Hospital (DTL-OXT-17).

### 2.2. Statistical Analysis

The sample size required to test the hypothesis on the difference in bone loss of the implants between the two groups was calculated. In the literature [12], we found that the average standard deviation of bone loss for implants placed at the crestal level was approximately 0.35 mm, while to obtain a significant difference with implant placed at the subcrestal level, it would be required to achieve a difference in bone loss of approximately 0.28–0.30 mm.

Following the standard procedure, to detect possible differences between treatments, we accepted a risk of 5% and a statistical power of 95, which produced a value of 30 implants in each comparison group.

The statistical significance of the results obtained was calculated using IBM SPSS Statistics 24.0 (International Business Machines Corp; New York, NY, USA). For qualitative variables, univariate logistic regressions, Chi-squared tests, and cross-checks between the variables were performed to determine the statistical significance of the differences. To determine the groups that made the difference, we used Haberman’s corrected standardised residuals, which allowed us to obtain the significance of the cells independently.

For quantitative variables, cross-checks were performed, and the normality test showed that not all variables analysed followed a normal distribution (Kolmogorov–Smirnov test). Therefore, the results of the corresponding non-parametric tests were considered for statistical significance, including Mann–Whitney U test for crossover with dichotomous variables or Kruskal–Wallis to determine the overall significance between the variables with more than two categories. In addition, when the test was significant, the Mann–Whitney U test was used for comparisons between the groups (two by two) to determine which groups were different from each other.

An association was considered statistically significant for values of *p* < 0.05 (*p* < 0.01, *p* < 0.001, *p* < 0.0001, and *p* < 0.00001), whereby the lower the figure, the greater the significance.

## 3. Results

The total study sample consisted of a total of 218 implants, of which 97 (44.5%), 91 (41.7%), and 30 implants (13.8%) were placed at centres A, B, and C, respectively. The mean age of the patients analysed was 58.56 ± 10.12 years, among which patients younger than 55 years made up 31.2% of the sample, those between 55 and 64 years comprised 43.1% of the sample, and 25.7% were over 65 years of age. A higher frequency of women was observed (54.6%) than men (45.4%).

Table 1 shows the qualitative characteristics of the implants placed and their evolution over two years. A total of 119 implants (54.6%) were placed in the upper jaw, of which 26.1% were in the anterior and 28.4% were in the posterior sector. A total of 99 implants (45.4%) were placed in the mandibular arch, of which 19.7% were in the anterior and 25.7% were in the posterior sector. In relation to the implant model used, 39 internal-conical-type implants (17.9%), 133 external-conical-type implants (61.6%), and 46 external-cylindrical-type implants (21.1%) were placed. The mean implant diameter was 3.94 ± 0.25 mm, and the mean implant length was 12.61 mm. In relation to the type of bone found, the highest percentage was type D2 (49.5%), followed by type D3 (30.7%). When the type of attachment used was analysed, 78% of the implants placed were straight transepithelial. Of the cases restored in the study, 88.5% were complete rehabilitations. In 93.1% of the implants inserted, the torque was higher than 35 N/cm; crestal expansion surgical torque was not performed in any of the cases analysed. At 24 months after implant surgery, a success rate of 98.2% was observed. Implant failure occurred within 2 months after implant insertion in three cases due to mobility, and in one case due to peri-implant infection.

Table 2 shows the quantitative characteristics of the implants placed and their evolution. A difference in the ISQ between osseointegration and the initial value after implant placement of 6.54 ± 7.74 was observed. The mean marginal bone loss observed at 2 months after prosthetic loading was 0.09 ± 0.20 mm; at 6 months, 0.20 ± 0.26 mm; at 12 months, 0.45 ± 0.41 mm; and at 24 months, 0.65 ± 0.59 mm. A mean marginal bone loss difference of 0.56 ± 0.60 mm was observed between 2 months and 24 months after prosthetic loading.

Table 3 and Table 4 shows the evolution of marginal bone loss between 2 and 6 months, 2 and 12 months, and 2 and 24 months, according to the variables analysed. For the last period, statistically significant differences were observed between marginal bone loss and the centre where the implant was placed (*p* < 0.0001). In relation to gender, a greater difference in marginal bone loss was observed in women (*p* < 0.0001). Greater marginal bone loss was observed in patients between 55 and 64 years of age (*p* < 0.0001), and according to the implant model, a greater difference was observed in external cylindrical implants (*p* < 0.0001). The diameter of the implant was greater in implants with a diameter greater than 4 mm (*p* < 0.0001), and according to the type of bone, a greater difference was observed in type D1 (*p* < 0.01). The type of attachment revealed a greater difference in bone loss in implants with straight transepithelial implants (*p* < 0.0001). Finally, when analysing the type of rehabilitation, a greater difference in marginal bone loss was observed in complete rehabilitations (*p* < 0.05) and implants in which the insertion torque was less than 35 N/cm (*p* < 0.01).

## 4. Discussion

Marginal bone loss is encompassed within a series of criteria used to define peri-implantitis or peri-implant disease, in which unfavourable changes occur at the level of the soft and bony tissues surrounding the dental implant once it is placed and subjected to the masticatory load [7,13].

A multitude of radiographic means were employed for the assessment of marginal bone loss (periapical radiography, orthopantomography, or cone beam tomography) [14,15,16,17]. In this study, the technique of periapical radiography with a paralleliser was used, in agreement with previously published studies. Other authors have even studied different digital protocols for measuring marginal bone loss [18].

A statistically significant increase in marginal bone loss was observed with increasing age [17]. Other authors have found greater marginal bone loss with age in men compared to women, although they observed a statistically significant point increase in women aged 50–60 years (*p* < 0.001) [19]. In this study, a greater difference in marginal bone loss was found between the ages of 50–60 years (*p* < 0.0001), and more so in women than in men (*p* < 0.0001), 2–24 months after prosthetic loading, which is in agreement with the previous study.

Multiple authors have found no statistically significant marginal bone loss differences due to the position in the arch in which the implant was placed, including the maxilla, mandible, anterior zone, and posterior zone [20,21,22]. In the study, no statistically significant differences were observed between implant location and marginal bone loss. These results differ from those found by other authors who have observed greater marginal bone loss in implants placed in the maxilla than in the mandible [19].

In relation to implant shape, a previous study found a statistically significant difference between marginal bone loss and the shape of cylindrical (0.88 ± 0.43 mm) and conical (0.61 ± 0.34 mm) implants, with a higher marginal bone loss observed in cylindrical implants (*p* < 0.05) [23]. These results coincide with those obtained in this study, in which a difference in marginal bone loss of 1.12 ± 0.32 mm was observed in cylindrical implants with respect to conical implants, where this was 0.41 ± 0.56 mm (*p* < 0.0001) between 2 and 24 months after prosthetic loading.

In relation to implant connection, previous studies have commented that there are no statistically significant differences associating marginal bone loss with either an internal or external implant connection [24,25]. In this study, a greater difference in the mean marginal bone loss between 2 and 24 months after prosthetic loading was found in externally connected implants (0.64 ± 0.62 mm) than internally connected implants (0.17 ± 0.24 mm) (*p* < 0.0001). These data are consistent with other studies published in the literature [26,27].

In relation to the implant diameter, previous studies have found an association between larger-diameter implants and greater marginal bone loss [19,28]. In this study, a greater difference in the mean marginal bone loss was observed in implants larger than 4 mm in diameter at 2–24 months after prosthetic loading, which is consistent with the previously mentioned studies. However, other authors have found no statistically significant association [21,29,30,31,32].

Numerous authors have analysed whether the length of the implant influences marginal bone loss, finding no statistically significant differences [28,33,34,35,36,37]. In this study, no statistically significant differences were found between implant length and marginal bone loss. However, other authors have found significant differences between the two variables [16].

A review of the literature shows that numerous authors have not drawn a significant association between bone type or quality and marginal bone loss [28,29,30,38]. In this study, a greater difference in marginal bone loss was observed in bones of type 1 quality.

In relation to the type of attachment, some authors have observed no significant differences in marginal bone loss between the use of a curved or straight abutment after one year of follow-up [39]. In this study, a greater difference in marginal bone loss was observed between 2 and 24 months after prosthetic loading in implants with a straight transepithelial (*p* < 0.0001). Some authors have found greater marginal bone loss when the definitive abutment was not placed after surgery [21,40,41]. In this study, the final abutment was not placed until the final placement of the prosthetic rehabilitation, which could have influenced the marginal bone loss.

In relation to surgical technique, previous studies have found no statistically significant differences in marginal bone loss between performing a mucoperiosteal flap prior to the dental implant placement [42,43]. In this study, all implants were placed after a mucoperiosteal flap.

The type of prosthetic rehabilitation has been considered an influential factor in marginal bone loss [17]. In this study, a greater difference in marginal bone loss was observed between 2 and 24 months after prosthetic loading in implants rehabilitated with complete restorations compared to partial or single restorations. However, other authors have not found statistically significant differences among these variables [21,41].

Primary stability after implant placement undergoes variations during the healing process [44]. In this study, an increase in the ISQ was observed between implant placement and after the osseointegration process, which corroborates the statements previously made [45].

Several authors have found no statistically significant differences in marginal bone loss in relation to the insertion torque [38,46]. In this study, it was observed that in implants in which the insertion torque did not exceed 35 N/cm, the difference in marginal bone loss was greater at 2–24 months after prosthetic loading than in implants in which this torque was exceeded. These results contrast those found by other authors, who have observed that when the insertion torque is lower, there is less marginal bone loss than when the insertion torque increases during dental implant placement [38,46].

Among the limitations that we have highlighted in our study, there was an inherent weakness of multicentre studies since they require additional control from all research centres. Likewise, the application of a two-dimensional radiological evaluation method limits our study, although it is a widely validated methodology used by current authors [8,9,10].

The measurement of the initial keratinised gingiva that the patients presented, as well as the milling applied, is an aspect that could also have been controlled and would have yielded interesting data, as has been pointed out in some studies [11,47].

Finally, and before moving on to the conclusion, where we will summarise the key results in relation to the objectives of our study, we should point out that the possible biases of our study, discussed above, can operate in either direction, given the multitude of controlled variables (despite the fact that all the measurements in the study have been double-checked by two different people) and the difficulties of conducting a multicentre study.

On the contrary, conducting a multicentre study with several surgeons and rehabilitators provides the results of our study with a strong external validity, and its results could easily be extrapolated to routine practice.

## 5. Conclusions

According to the results obtained in this study, we can indicate that marginal bone loss is sensitive to multiple factors, such as the prosthetic connection, age, gender, implant width, bone density, type of rehabilitation, and torque insertion. However, the length of the implant did not seem relevant to the peri-implant bone loss studied within a two-year follow-up of our sample. However, we must be cautious in assuming these results, given the limitations of the study, the multiplicity of analyses, and other issues discussed above.

## Figures and Tables

**Table 1:** Characteristics of the implants and their evolution (qualitative).

		n	%
Maxillary—mandibular position	Maxillary	119	54.6
	Mandibular	99	45.4
Anteroposterior position	Anterior	100	45.9
	Posterior	118	54.1
“Crossed” position	Maxillary—anterior	57	26.1
	Maxillary—posterior	62	28.4
	Mandibular—anterior	43	19.7
	Mandibular—posterior	56	25.7
Implant model	Internal conical	39	17.9
	External conical	133	61.0
	Internal cylindrical	0	0.0
	External cylindrical	46	21.1
Diameter	Up to 4 mm	197	90.4
	More than 4 mm	21	9.6
		Mean	S.D.
		3.94	0.25
Length	Up to 10 mm	24	11.0
	More than 10 mm	194	89.0
		Mean	S.D.
		12.61	1.50
Bone type	D1	41	18.8
	D2	108	49.5
	D3	67	30.7
	D4	2	0.9
Type of prosthetic connection	Direct to implant	39	17.9
	Transepithelial straight	170	78.0
	Transepithelial angled	9	4.1
	Multi-unit	0	0.0
Restoration type	Full	193	88.5
	Partial	13	6.0
	Unitary	12	5.5
Torque > 35 N/cm	Yes	203	93.1
	No	15	6.9
Crestal expansion	Yes	0	0.0
	No	218	100.0
Failure (2 months)	Yes	4	1.8
	No	214	98.2
Cause of failure (2 months)	Mobility	3	75.0
	Peri-implant infection	1	25.0
Failure (6 months)	Yes	4	1.8
	No	214	98.2
Cause of failure (6 months)	Mobility	3	75.0
	Peri-implant infection	1	25.0
Failure (12 months)	Yes	4	1.8
	No	214	98.2
Cause of failure (12 months)	Mobility	3	75.0
	Peri-implant infection	1	25.0
Failure (24 months)	Yes	4	1.8
	No	214	98.2
Cause of failure (24 months)	Mobility	3	75.0
	Peri-implant infection	1	25.0

**Table 2:** Characteristics of the implants and their evolution (quantitative).

Variable	No.	Mean	S.D.	Normality
ISQ initial value	97	74.55	7.96	Yes
ISQ osseointegration value	90	80.81	5.77	No
ISQ difference (osseointegration—initial)	90	6.54	7.74	Yes
Bone loss at two months (mm)	214	0.09	0.20	No
Bone loss at six months (mm)	214	0.20	0.26	No
Bone loss at 12 months (mm)	214	0.45	0.41	No
Bone loss at 24 months (mm)	214	0.65	0.59	No
Bone loss difference (2–6 months) (mm)	214	0.11	0.25	No
Bone loss difference (2–12 months) (mm)	214	0.36	0.42	No
Bone loss difference (2–24 months) (mm)	214	0.56	0.60	No
Bone loss difference (6–12 months) (mm)	214	0.25	0.38	No
Bone loss difference (6–24 months) (mm)	214	0.45	0.55	No
Bone loss difference (12–24 months) (mm)	214	0.20	0.30	No

**Table 3:** Evolution of marginal bone loss by time periods according to the variables analysed (mean and SD).

	Bone Loss m	2–6 Months		2–12 Months		2–24 Months	
Variable		Mean	Standard Deviation	Mean	Standard Deviation	Mean	Standard Deviation
Sample		0.11	0.25	0.36	0.42	0.56	0.60
Gender							
Female		0.14	0.24	0.47	0.43	0.74	0.61
Male		0.07	0.25	0.22	0.37	0.34	0.51
*p*		<0.05		<0.0001		<0.0001	
Age							
Under 55		0.06	0.24	0.14	0.41	0.25	0.56
From 55 to 64 years		0.15	0.27	0.47	0.42	0.74	0.57
65 or over		0.09	0.19	0.43	0.35	0.63	0.55
*p*		-		<0.0001		<0.0001	
Location							
Maxillary—anterior		0.12	0.23	0.38	0.41	0.57	0.58
Maxillary—posterior		0.12	0.31	0.33	0.42	0.49	0.58
Mand—anterior		0.12	0.22	0.42	0.41	0.65	0.57
Mand—posterior		0.08	0.19	0.32	0.45	0.55	0.67
*p*		-		-		-	
Implant Model							
Internal conical		0.07	0.17	0.14	0.23	0.17	0.24
External conical		0.10	0.25	0.29	0.42	0.48	0.61
External cylindrical		0.17	0.26	0.73	0.33	1.12	0.32
*p*		-		<0.0001		<0.0001	
Implant Diameter							
Up to 4 mm		0.10	0.24	0.32	0.42	0.49	0.58
More than 4 mm		0.19	0.25	0.69	0.33	1.21	0.30
*p*		-		<0.001		<0.0001	
Implant Length							
Up to 10 mm		0.21	0.33	0.44	0.54	0.71	0.64
More than 10 mm		0.10	0.23	0.35	0.41	0.54	0.59
*p*		<0.05		-		-	
Bone Type							
D1		0.08	0.18	0.46	0.43	0.75	0.62
D2		0.11	0.23	0.28	0.39	0.43	0.57
D3		0.11	0.29	0.42	0.45	0.65	0.60
*p*		-		<0.05		<0.01	
Type Of Prosthetic Connection							
Direct to implant		0.00	0.26	0.03	0.41	0.11	0.58
Transepithelial straight		0.13	0.23	0.43	0.40	0.67	0.57
Transepithelial angled		0.22	0.26	0.33	0.25	0.33	0.25
*p*		<0.01		<0.0001		<0.0001	
Type of Rehabilitation							
Full		0.12	0.23	0.38	0.41	0.59	0.59
Partial		−0.04	0.40	0.25	0.69	0.50	0.85
Unitary		0.04	0.14	0.08	0.19	0.08	0.19
*p*		-		<0.05		<0.05	
Insertion Torque > 35 N/cm							
Yes		0.10	0.23	0.34	0.42	0.53	0.58
No		0.31	0.33	0.62	0.46	1.04	0.63
*p*		<0.01		<0.05		<0.01	

**Table 4:** Evolution of marginal bone loss by time period according to the variables analysed (median and IQ).

	Bone Loss m	2–6 Months		2–12 Months		2–24 Months	
Variable		Median	IQ	Median	IQ	Median	IQ
Sample		0.00	[0.00–0.00]	0.50	[0.00–0.50]	0.50	[0.00–1.00]
Gender							
Female		0.00	[0.00–0.50]	0.50	[0.00–1.00]	1.00	[0.00–1.25]
Male		0.00	[0.00–0.00]	0.00	[0.00–0.50]	0.00	[0.00–1.00]
*p*		<0.05		<0.0001		<0.0001	
Age							
Under 55		0.00	[0.00–0.00]	0.00	[0.00–0.50]	0.00	[0.00–0.50]
From 55 to 64 years		0.00	[0.00–0.50]	0.50	[0.00–1.00]	1.00	[0.00–1.00]
65 or over		0.00	[0.00–0.00]	0.50	[0.00–0.50]	0.50	[0.00–1.00]
*p*		-		<0.0001		<0.0001	
Location							
Maxillary—anterior		0.00	[0.00–0.38]	0.50	[0.00–0.50]	0.50	[0.00–1.00]
Maxillary—posterior		0.00	[0.00–0.50]	0.50	[0.00–0.50]	0.50	[0.00–1.00]
Mand—anterior		0.00	[0.00–0.13]	0.50	[0.00–1.00]	0.75	[0.00–1.00]
Mand—posterior		0.00	[0.00–0.00]	0.00	[0.00–0.50]	0.50	[0.00–1.00]
*p*		-		-		-	
Implant Model							
Internal conical		0.00	[0.00–0.00]	0.00	[0.00–0.50]	0.00	[0.00–0.50]
External conical		0.00	[0.00–0.00]	0.00	[0.00–0.50]	0.50	[0.00–1.00]
External cylindrical		0.00	[0.00–0.50]	1.00	[0.50–1.00]	1.00	[1.00–1.50]
*p*		-		<0.0001		<0.0001	
Implant Diameter							
Up to 4 mm		0.00	[0.00–0.00]	0.00	[0.00–0.50]	0.50	[0.00–1.00]
More than 4 mm		0.00	[0.00–0.50]	0.50	[0.50–1.00]	1.00	[1.00–1.50]
*p*		-		<0.001		<0.0001	
Implant Length							
Up to 10 mm		0.00	[0.00–0.50]	0.50	[0.00–1.00]	1.00	[0.13–1.00]
More than 10 mm		0.00	[0.00–0.00]	0.50	[0.00–0.50]	0.50	[0.00–1.00]
*p*		<0.05		-		-	
Bone Type							
D1		0.00	[0.00–0.00]	0.50	[0.00–1.00]	1.00	[0.00–1.38]
D2		0.00	[0.00–0.00]	0.00	[0.00–0.50]	0.00	[0.00–1.00]
D3		0.00	[0.00–0.50]	0.50	[0.00–1.00]	0.75	[0.00–1.00]
*p*		-		<0.05		<0.01	
Type Of Prosthethic Connection							
Direct to implant		0.00	[0.00–0.00]	0.00	[0.00–0.00]	0.00	[0.00–0.00]
Transepithelial straight		0.00	[0.00–0.50]	0.50	[0.00–0.95]	0.50	[0.00–1.00]
Transepithelial angled		0.00	[0.00–0.50]	0.50	[0.00–0.50]	0.50	[0.00–0.50]
*p*		<0.01		<0.0001		<0.0001	
Type of Rehabilitation							
Full		0.00	[0.00–0.50]	0.50	[0.00–0.50]	0.50	[0.00–1.00]
Partial		0.00	[0.00–0.00]	0.25	[−0.38–1.00]	1.00	[−0.38–1.00]
Unitary		0.00	[0.00–0.00]	0.00	[0.00–0.00]	0.00	[0.00–0.00]
*p*		-		<0.05		<0.05	
Insertion Torque > 35 N/cm							
Yes		0.00	[0.00–0.00]	0.50	[0.00–0.50]	0.50	[0.00–1.00]
No		0.50	[0.00–0.50]	1.00	[0.00–1.00]	1.50	[0.50–1.50]
*p*		<0.01		<0.05		<0.01	

## Data Availability

The research data will be accessible upon request to the authors.
